# INSIGHT (Staff): Prison Healthcare Professionals’ Attitude to Medical Student Psychiatry Placements and Understanding of Social Determinants of Health

**DOI:** 10.1192/bjo.2025.10298

**Published:** 2025-06-20

**Authors:** Sadia Nafees, Arthur Jones, Andrea Taylor-Clutton, Justin Lawson, Rob Poole

**Affiliations:** 1 Bangor University, Wrexham, United Kingdom; 2 BCUHB, Wrexham, United Kingdom; 3 Cardiff Medical School, Cardiff, United Kingdom; 4 Gables Medical (Offender Health) Ltd, Wrexham, United Kingdom

## Abstract

**Aims:** The Social Determinants of Health (SDOH) are generally taught as epidemiological facts rather than clinically relevant context. Psychiatry placements at HMP Berwyn (INSIGHT) provide medical students with exposure to SDOH at an individual level, allowing them to learn about the unique challenges faced by both the inmates and clinicians.

**Aims were to** explore healthcare professionals’ (HCPs) attitudes on psychiatry prison placements as a medical education measure to teach about social determinants in physical and mental health.

**Methods:** HCPs working at HMP Berwyn were surveyed. Questions were structured to answer whether these placements benefit students, improve their understanding of SDOH, and whether having students present is disruptive.

**Results:** We collected 25 out of 42 (60%) responses in Mar 2024. Key results include: 75% of respondents strongly agreed that psychiatry placements in prison are beneficial for medical students; 71% believed the prison placements should be continued as part of the medical education; 54% indicated that students had a poor understanding of SDOH at the start of the placement; 42% agreed that students’ understanding of SDOH improved by the end of their placements; and 42% strongly disagreed that having students in prison was disruptive.

**Discussion:** The majority of the participants viewed psychiatry placements in prison positively, emphasising their role in enhancing students’ understanding of SDOH. Additionally, most of the staff did not find the placements disruptive, supporting the continuation or extension of the programme. Potential limitations include response bias from participants with strong opinions and the absence of time-dependent data. Our ongoing research will explore student experiences and track staff opinions over time.

**Conclusion:** In our previous publications during the past three years, we have highlighted students’ positive responses to prison placements. This study further demonstrates that the HCPs at HMP Berwyn support these placements, recognising their value in improving students’ understanding of SDOH in challenging environments. This awareness exposes the importance of in-depth patient history-taking and equips future doctors to approach patient care holistically, ultimately fostering more equitable healthcare delivery and improving patient outcomes.

